# Evaluation of artificial intelligence model for crowding categorization and extraction diagnosis using intraoral photographs

**DOI:** 10.1038/s41598-023-32514-7

**Published:** 2023-03-30

**Authors:** Jiho Ryu, Ye-Hyun Kim, Tae-Woo Kim, Seok-Ki Jung

**Affiliations:** 1 Department of Orthodontics, School of Dentistry, Dental Research Institute, Seoul National University, 101 Daehak-ro, Jongno-gu, Seoul, 03080 Republic of Korea; 2 Department of Orthodontics, Korea University Guro Hospital, 148 Gurodong-ro, Guro-gu, Seoul, 08308 Republic of Korea

**Keywords:** Oral diseases, Computer science

## Abstract

Determining the severity of dental crowding and the necessity of tooth extraction for orthodontic treatment planning are time-consuming processes and there are no firm criteria. Thus, automated assistance would be useful to clinicians. This study aimed to construct and evaluate artificial intelligence (AI) systems to assist with such treatment planning. A total of 3,136 orthodontic occlusal photographs with annotations by two orthodontists were obtained. Four convolutional neural network (CNN) models, namely ResNet50, ResNet101, VGG16, and VGG19, were adopted for the AI process. Using the intraoral photographs as input, the crowding group and the necessity of tooth extraction were obtained. Arch length discrepancy analysis with AI-detected landmarks was used for crowding categorization. Various statistical and visual analyses were conducted to evaluate the performance. The maxillary and mandibular VGG19 models showed minimum mean errors of 0.84 mm and 1.06 mm for teeth landmark detection, respectively. Analysis of Cohen’s weighted kappa coefficient indicated that crowding categorization performance was best in VGG19 (0.73), decreasing in the order of VGG16, ResNet101, and ResNet50. For tooth extraction, the maxillary VGG19 model showed the highest accuracy (0.922) and AUC (0.961). By utilizing deep learning with orthodontic photographs, dental crowding categorization and diagnosis of orthodontic extraction were successfully determined. This suggests that AI can assist clinicians in the diagnosis and decision making of treatment plans.

## Introduction

Malocclusion is a condition characterized by malpositioned dental and skeletal components related to various local and systemic factors that can either directly or indirectly cause not only mastication, pronunciation, swallowing, and esthetic problems but also dental caries, facial growth impairment, and lower quality of life^1^. The etiology of malocclusion is multifactorial, and any genetic, environmental, or combined factors can be accountable, which could lead to dental caries, eruption problems, ankylosis, oral habits, trauma, etc.^2,3^ There could be many other factors that cause crowding affected by the malpositioning of teeth; however, dental crowding usually results from space deficiency when the available basal space is smaller than that required^4^. To resolve such malocclusion and crowding originating from space discrepancy, various degrees of orthodontic treatment can be planned, from simple minor tooth movement to orthognathic surgery^5^.

If the crowding mainly results from poor teeth positioning, it can be treated with a movement of the teeth, while there is a possibility that tooth extraction for making sufficient space is necessary when all teeth cannot be appropriately aligned on the basal arch^6^. However, orthodontic treatments accompanying tooth extractions are invasive and irreversible. Furthermore, the overall treatment time for orthodontic treatment, which usually ranges from 18 to 30 months, can be prolonged if tooth extractions are included^7^. Therefore, determining the severity of dental crowding and the decision to perform orthodontic extraction are essential factors for planning orthodontic treatment in terms of time and managing irreversibility.

The orthodontic treatment planning procedure includes taking various clinical materials from patients, for example, X-rays such as lateral cephalograms, posteroanterior cephalograms, panoramic radiographs, periapical radiographs, maxillary and mandibular study models, and facial and intraoral photographs^8^. Some of these methods have evolved with technical improvements in that digital radiographic and photographic procedures have replaced analog procedures. Furthermore, dental study casts can be converted to digital three-dimensional data using extraoral scanners, and even direct intraoral scanning of patients is possible nowadays^9^. All these materials from patients provide either exclusive or complementary information, enabling the establishment of better diagnoses and treatment plans^10^. Among the clinical materials, intraoral photographs are not only non-invasive and non-radioactive but can also be stored in a long-term manner such that clinicians can inspect the patient’s intraoral problems in detail with sufficient time.

Since artificial intelligence (AI) has gained tremendous popularity, the era of artificial intelligence has also come to the field of dentistry. In the orthodontics area, the decision of orthodontic tooth extraction, which has no firm consensus of criteria, has been long researched to find automated algorithms^11–14^, and many different subjects have been studied, for instance, the need for orthognathic surgery^15–17^, landmark detection on two- and three-dimensional cephalograms^18–20^, determination of skeletal malocclusion^21^, automated classification of clinical orthodontic photos^22^, and segmentation and labeling of teeth^23^ using deep learning techniques.

Artificial intelligence, in broad terms, as a research area, can be described as creating automated systems to perform tasks and solve problems without specific rules but by learning the data as humans do. Among the various subfields, convolutional neural network (CNN) deep learning techniques have been widely used in image processing^24^ and have also become popular in the medical and dental fields^25–27^. CNNs mimic the mechanism of human neurons as layers to calculate a large number of arithmetic equations to detect or classify the target images^27^ and the parameters or weights are automatically adjusted and modified during the learning process^28^. A study has even shown that clinical diagnosis using deep learning can classify skin diseases as accurately as medical specialists^29^.

One of the traditionally and widely used methods for the numerical analysis of dental crowding is the calculation of arch length discrepancy (ALD). If the ALD value indicates a space shortage, which means that the required space for normal dental alignment is larger than the available space, the dentition will be classified as “crowding” because there is not enough space for all teeth to be properly positioned. In this case, teeth extraction might be needed. On the other hand, if the available space is larger than the required space, the dentition will be considered “spacing” due to the redundant space among teeth^30^. In orthodontic treatment planning, there are many factors that could affect the extraction decision, such as systemic diseases, remaining growth, and patients’ chief complaints; thus, the sole ALD value cannot be the absolute criterion for extraction but should be one of the prior criteria when de-crowding is required^31,32^. With the given normal ALD value, tooth extraction might be required when treating bimaxillary protrusion, resolving midline discrepancy, improving profile outline, involving orthognathic surgery, and other esthetic considerations are important^33^.

Orthodontists make treatment plans for patients not only by clinical materials but also by cumulated experiences and possible bias from previous treatment outcomes. This means that treatment plans can be affected by the clinician’s personal experiences, background, philosophy, esthetic standards, and affiliated school^34^. Consequently, the decision on whether extraction should be involved is both objective and subjective. In a clinical situation, the extraction decision is usually made using several clinical materials; however, it can also be made using only orthodontic intraoral photographs because they contain sufficient information for experienced clinicians. In this manner, orthodontic specialists could determine the necessity of extractions using clinical photos, and therefore, AI could determine it by training on the accumulated data.

The aim of this study was to set up artificial intelligence models for tooth landmark detection and tooth extraction diagnosis using feeding data from clinical digital intraoral photographs that are routinely taken for orthodontic treatment planning and then categorize crowding severity by calculating arch length discrepancy, followed by analyzing the accuracy of deep learning models in various ways.

## Materials and methods

### Dataset and data preparation

The entire dataset used in this study was obtained from the Seoul National University Dental Hospital Intranet database. It consists of clinical photographs and metadata of patients who visited the Department of Orthodontics and had intraoral photographs taken for orthodontic diagnoses. After excluding photographs of poor quality, severe focus problems, any loss or unerupted permanent teeth, and having orthodontic appliances or retainers, 1500 maxillary and 1636 mandibular individual intraoral photos were extracted from a total of 1636 patients (786 males and 850 females). This study was conducted according to the guidelines of the Declaration of Helsinki, and approved by the appropriate Institutional Review Board of Seoul National University Dental Hospital (ERI19036). All methods were performed in accordance with the relevant guidelines and regulations.

The mean ages of patients were 26.3 and 23.7 years old and standard deviations were 4.2 and 5.3 for male and female samples, respectively. Each photograph object was assigned a unique identification number, and any personally identifiable information was removed, except for the patient’s age.

Two orthodontic specialists together annotated the conformed mesial and distal points of each tooth and whether the teeth should be extracted for all images. The following coordinates were manually identified: mesial and distal points of the left and right central incisors, lateral incisors, canines, first premolars, second premolars, and a single central point of the basal arch form. The central point of the basal arch form is defined as the midpoint of the whole arch shape both horizontally and antero-posteriorly. There were no final disagreements among the results. All resulting data were considered as the ground truth for future comparative analysis.

For the learning and testing processes, the objects were split into learning and test datasets without duplication, using a random function in Microsoft Excel software. The test dataset consisted of 200 maxillary and 200 mandibular photographs, and the learning dataset comprised 1,300 maxillary and 1,436 mandibular photographs without overlapping (Table 1). The photographs in the test dataset were used only during the testing process.

**Table 1:** The characteristics of the photographs used in this study.

	Non-extraction	Extraction	Total
Learning set			
Maxilla	1053	247	1300
Mandible	1152	284	1436
Test set			
Maxilla	163	37	200
Mandible	155	45	200

To improve the performance of deep learning models with a certain number of original samples, a data augmentation technique^35^ was utilized with caution to avoid degrading the diagnostic characteristics of each photograph. For augmentation methods, affine transformations of random window cropping and color modifications, including gamma, brightness, and contrast adjustment, were used.

### Landmark detection and crowding categorization model

**Figure 1:**
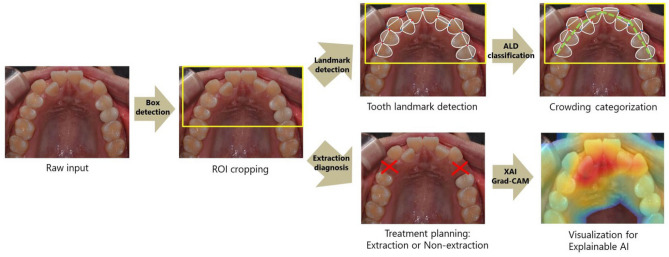
Overall flow diagram of the processes: the input photographs initially undergo ROI cropping and are then fed into the models for landmark detection, crowding categorization, and tooth extraction diagnosis.

In this study, two artificial intelligence models—with photograph input data—were constructed for different purposes: landmark detection models with a crowding categorization function and orthodontic extraction diagnosis models (Fig. 1). The landmark detection program can identify the central point of the basal arch form and each tooth’s mesial and distal points, followed by calculating the ALD for dental crowding categorization.

**Figure 2:**
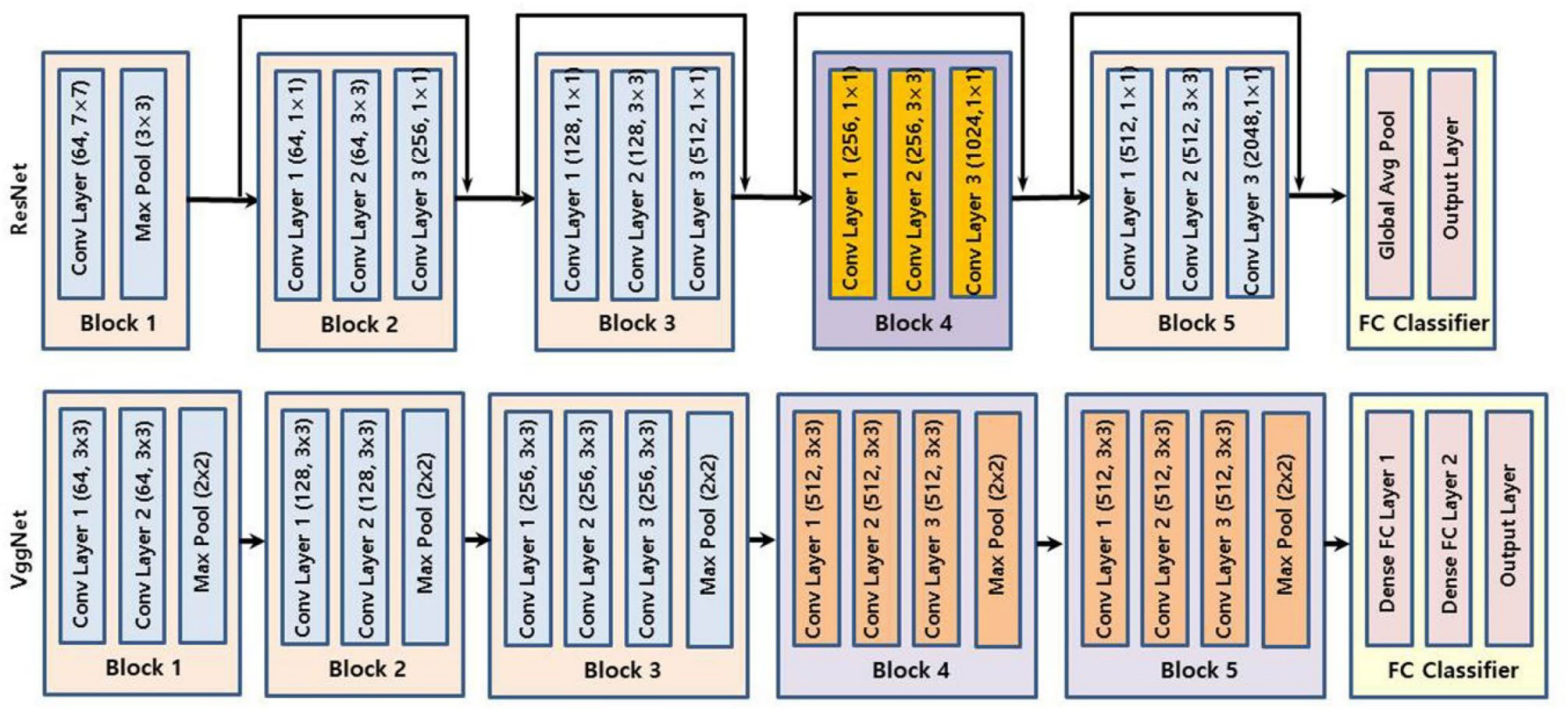
Basic structures of ResNet and VggNet models. By utilizing skip connections in ResNet, the degradation problem can be diminished. VggNet has simple structures such that the depth of the network can be increased to achieve better performance.

First, rectangular-shaped region of interest (ROI) windows were extracted from the feeding images using a Faster R-CNN model. Landmark detection of the cropped images was then performed with ResNet^36^ and VggNet^37^ models, printing the location of each point. Finally, using the output coordinates, dental crowding categorization was processed and output. These AI models have been used in many image classification studies with high success rates, and their basic structures are publicly available (Fig. 2). In this study, two specific models with different layer depths in each network model were used to compare the performance according to the layer depth: ResNet50 and ResNet101 for ResNet and VGG16 and VGG19 for VggNet.

The arch length discrepancy value is traditionally defined as the difference between the basal arch length and the sum of the mesiodistal breadths of all incisors, canines, and premolars. If the value is above zero, meaning the basal arch length is larger than the corresponding dentitions, there is a redundant space leading to the crowding category “spacing.” By contrast, this means that there is a space shortage for the teeth to be well aligned if the value has a negative sign. In this study, the crowding category was defined by the ALD value as follows: “normal” for − 1 to 0, “mild crowding” for − 4 to − 1, “moderate crowding” for − 7 to − 4, and “severe crowding” for the rest of the negative values, all in millimeter units. While the categories were defined by an absolute length in millimeters, the digital photographs only had pixel unit information because there were no magnification data. Therefore, to convert the pixel lengths in the digital photos into relative millimeter lengths, the mesiodistal widths of the maxillary right central incisor as 8.41 mm and mandibular right central incisor as 5.26 mm in the photographs were set as standards. These mean values were calculated from a study on tooth and arch sizes in the Korean population^38^.

### Extraction diagnosis model

The same ResNet50, ResNet101, VGG16, and VGG19 were used as base models for tooth extraction diagnosis. Transfer learning and fine-tuning techniques were used for the learning process. For each model, the fully connected classifier part was modified to conform to the extraction diagnosis output. The ground truth of the extraction decision as a training parameter was fed as binary data, and the determination of whether the extraction was needed was output by the model as a decimal number from zero to one. While the value itself is a continuous number that approaches one as the necessity of extraction increases, the final diagnosis should be binary. Therefore, the Softmax activation function was used to make the output data either zero or one. As the extraction decision was predicted from the input image without any additional data, the gradient-weighted class activation mapping (Grad-CAM) technique^39^ was used to visualize which photo area the AI model was more interested in. Using this approach, an explainable AI model can be constructed by utilizing a heatmap plot to differentiate colors over the input image to show the region where the AI model decision was substantially affected.

### Model training and performance evaluation

The learning and test processes were performed using the Keras 2.3.1 framework for Python 3.7.4 with Tensorflow-GPU 2.5.0 backend on a Microsoft Windows 10 workstation with an Nvidia RTX A6000 (48 GB VRAM) GPU and 256 GB of RAM. Every image fed to the models was resized to 224 × 224 pixels to satisfy the model’s input format requirements. The training and validation datasets were divided into 8:2 proportions, without human intervention. All training procedures implemented a fivefold cross-validation process to handle the overfitting problem^40^, and the network parameters were updated using the Adam optimizer. The batch size and number of epochs were set to 256 and 150, respectively. The initial learning rate was set to 0.003, and the parameter was multiplied by 0.2 when the validation loss was not improved by 0.000001 during three consecutive epochs. The learning process was terminated prematurely if the validation loss did not improve by 0.000001 during eight successive epochs. The training accuracy, training loss, validation accuracy, and validation loss were measured and recorded for the entire learning process.

To compare the ground truth and AI-predicted locations of landmarks, the distance between two points of identical landmarks in the same photograph was calculated, and the mean error and 95% confidence interval were computed. Scattergrams and confusion matrices for visual comparison were obtained from the results, and Cohen’s weighted kappa coefficients were calculated for analytical performance evaluation. As the tooth extraction decision was similar to a true or false problem, the accuracy, sensitivity, and specificity values were calculated for the analysis of the models, and receiver operating characteristic (ROC) curves accompanied by the area under the ROC curve (AUC) value was drawn.

### Ethics approval and consent to participate

This study was approved by the Institutional Review Board of Seoul National University Dental Hospital (ERI19036). The informed consent was waived by the Institutional Review Board of Seoul National University Dental Hospital because the clinical photographs were taken for treatment use, and there was no identifiable patient information.

## Results

### Landmark detection and crowding categorization model

**Figure 3:**
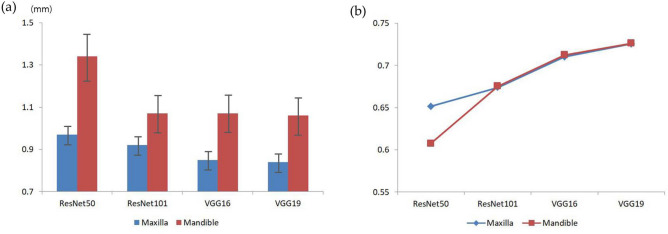
Mean errors and weighted kappa coefficients of landmark detection with crowding categorization. (**A**) The maxilla models consistently show smaller error values than the mandible ones. The mean errors decrease in the order of ResNet50, ResNet101, VGG16, and VGG19. (**B**) All kappa values suggest that the strength of agreement for crowding classification is substantial, and the agreement becomes higher as the layer becomes deeper in the same network models.

**Figure 4:**
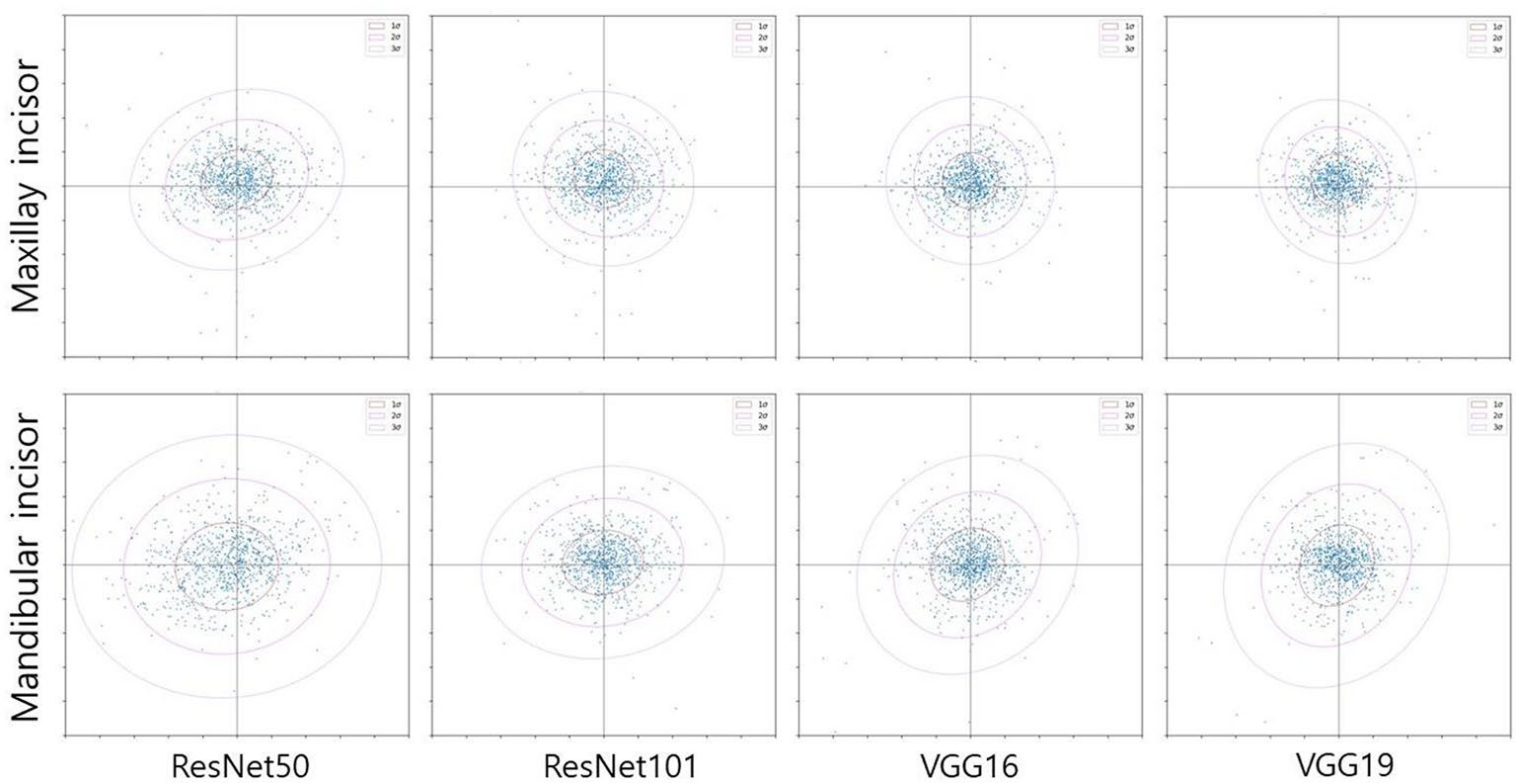
Maxillary and mandibular incisor scattergrams and confidence ellipses for the mean errors obtained from each landmark detection model. The maxillary models and ResNet network result in a more uniformly distributed pattern of errors than the mandibular models and VggNet.

**Figure 5:**
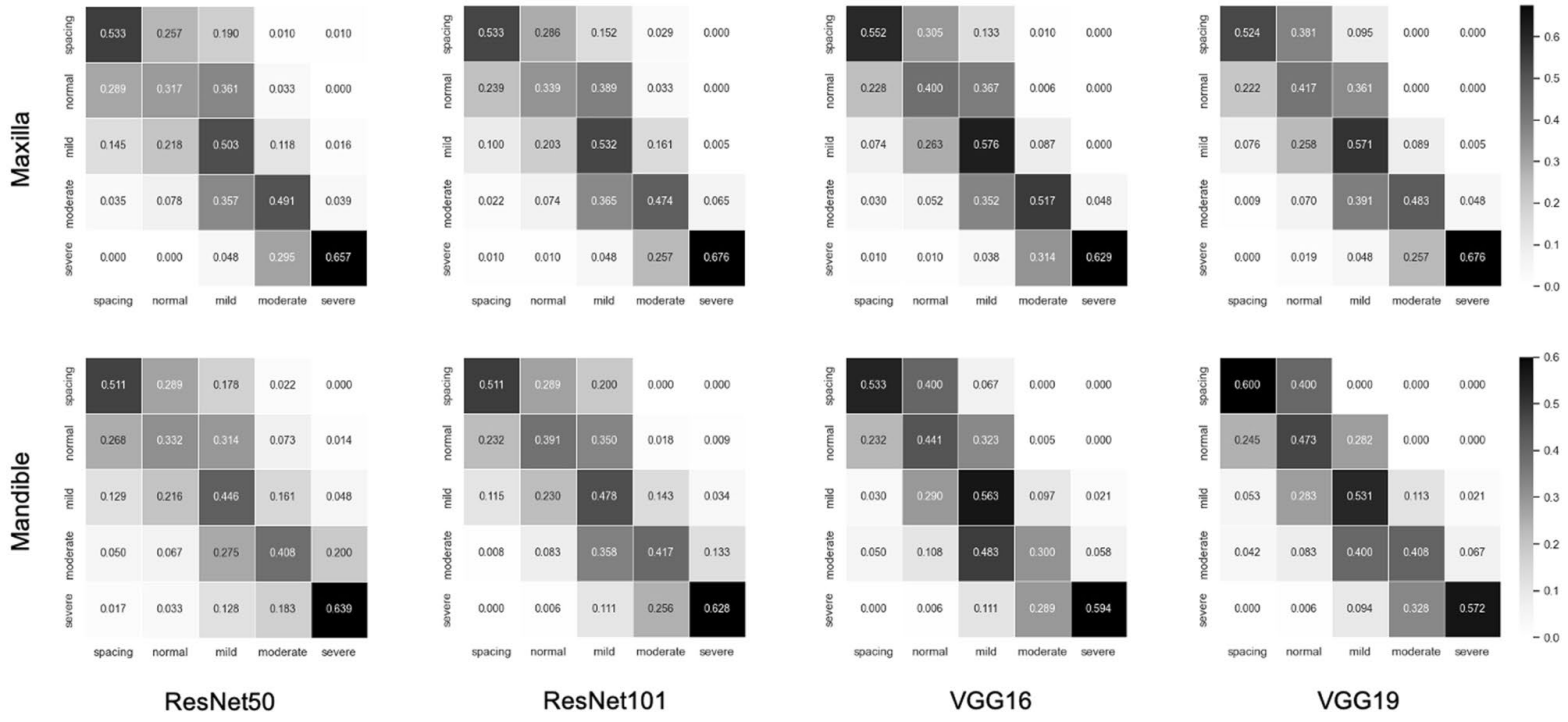
Normalized confusion matrices of the crowding categorization agreement. The diagonal pattern indicates that the agreement is relatively strong. They show higher accuracy for “spacing” and “severe crowding” as they are darker than the others.

The mean errors of the landmark points and weighted kappa values for crowding categorization are listed in Table 2. In the landmark detection analysis, the maxillary VGG19 model showed the smallest mean error value of 0.84 mm, whereas the mandibular ResNet50 model showed the largest mean error value of 1.34 mm. For both the maxilla and mandible, the ResNet50 model showed the largest mean errors, and the VGG19 model showed the smallest mean errors compared to other models. As shown in Fig. 3, the mean errors of the VggNet, maxilla, and deeper layer models were consistently smaller than those of the ResNet, mandible, and shallower layer models, respectively. Moreover, the plots indicate that the more accurate the landmark detection the more gain in uniformity (Fig. 4). In the crowding categorization analysis, the VGG19 model showed the highest Cohen’s weighted kappa value of 0.73 in both the maxilla and mandible, whereas the ResNet50 model showed the lowest kappa coefficients of 0.65 for the maxilla and 0.61 for the mandible. Similar to the landmark detection analysis, all the VggNet models had higher kappa values than ResNet, and the deeper layer models had higher kappa values. The confusion matrix plots (Fig. 5) were drawn for agreement tendency visualization, and they represent that the models could discern “spacing” and “severe crowding” among other categories better.

**Table 2:** Maxillary and mandibular absolute mean errors (mm) of landmark points with 95% CI and Cohen’s weighted kappa coefficients of crowding categorization.

Model	ResNet50	ResNet101	VGG16	VGG19
Maxilla				
Mean error	0.97	0.92	0.85	0.84
95% CI	0.92–1.02	0.87–0.97	0.80–0.90	0.79–0.89
Weighted Kappa	0.65	0.67	0.71	0.73
Mandible				
Mean error	1.34	1.07	1.07	1.06
95% CI	1.22–1.46	0.97–1.17	0.97–1.17	0.96–1.16
Weighted Kappa	0.61	0.68	0.71	0.73

*CI* confidence interval.

### Extraction diagnosis model

**Figure 6:**
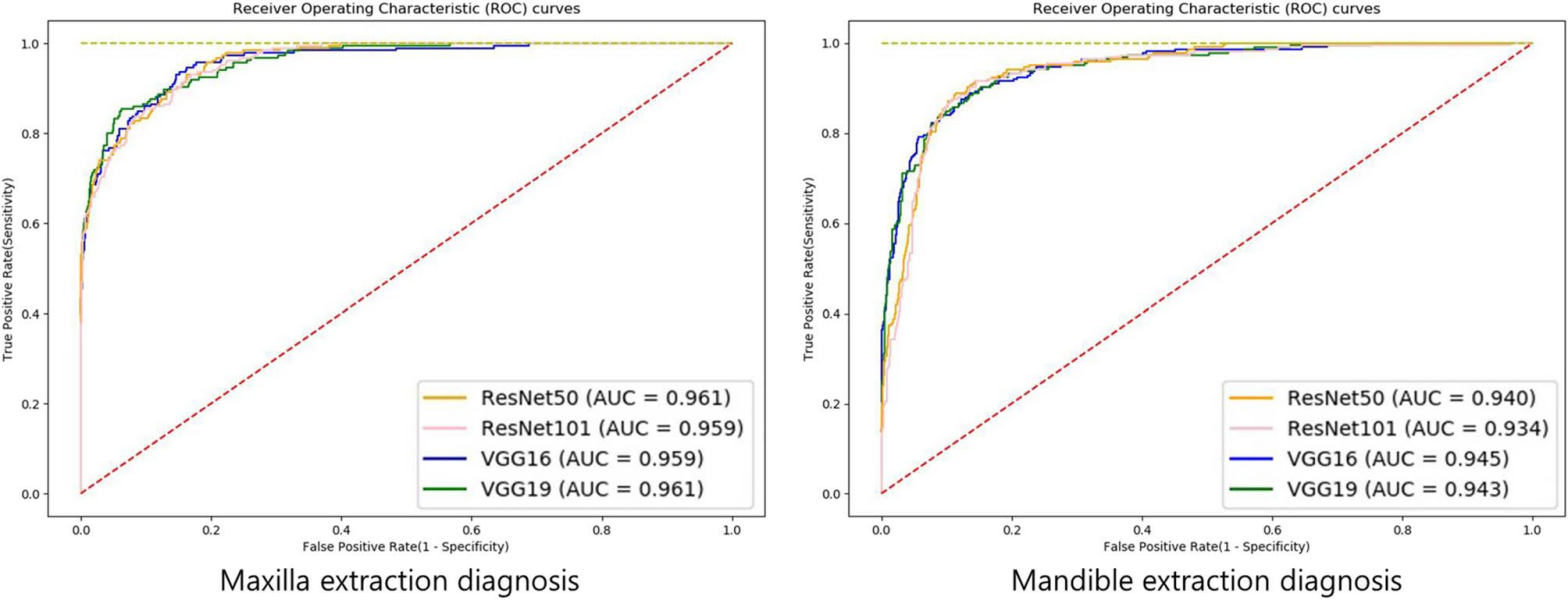
Receiver operating characteristic curves show that all models resulted in high accuracy.

Table 3 shows the accuracy, sensitivity, specificity, and AUC of each extraction diagnosis model. The ROC curves, which can be seen in Fig. 6, indicate the performance of the models in which the strength of agreement becomes higher when the lines approach the upper left corner. The AUC is a unitless value of the geometric area under each curve, which ranges from zero to one, where the value is one if there is a perfect agreement for all cases. The highest AUC value was 0.961 for the maxillary VGG19 model and the lowest AUC value was 0.934 for the mandibular ResNet101 model. Overall, the maxillary VGG19, mandibular VGG19, and VGG16 models generally had higher accuracy. Unlike the landmark detection model, the extraction diagnosis model did not show a clear performance tendency according to the model and depth of the network, except that the maxillary screening performance was higher than that of mandibular.

**Table 3:** Screening performance of tooth extraction diagnosis models.

Model	ResNet50	ResNet101	VGG16	VGG19
Maxilla				
Accuracy	0.909	0.915	0.910	0.922
Sensitivity	0.778	0.751	0.811	0.854
Specificity	0.939	0.952	0.933	0.937
AUC	0.961	0.959	0.959	0.961
Mandible				
Accuracy	0.895	0.890	0.898	0.898
Sensitivity	0.778	0.716	0.796	0.818
Specificity	0.929	0.941	0.928	0.921
AUC	0.940	0.934	0.945	0.943

## Discussion

The orthodontic treatment planning process is undoubtedly crucial, and various clinical data should be considered carefully in complicated ways, especially for irreversible dental procedures. In this process, as in other fields of medicine, qualitative and subjective opinions such as personal knowledge, specialty, experiences, and philosophy are involved^41^. These elements make it challenging to build an automated diagnosis algorithm using several conditional statements and mathematical formulas. Determining the amount of dental crowding through ALD measurement on stone casts to recognize how much space should be gained and deciding whether to perform tooth extractions as a surgical aspect is rather complicated and demands high concentration. This study demonstrated the possibility of a clinically reliable automated crowding categorization and orthodontic extraction diagnosis system without any actual stone models or additional materials.

**Figure 7:**
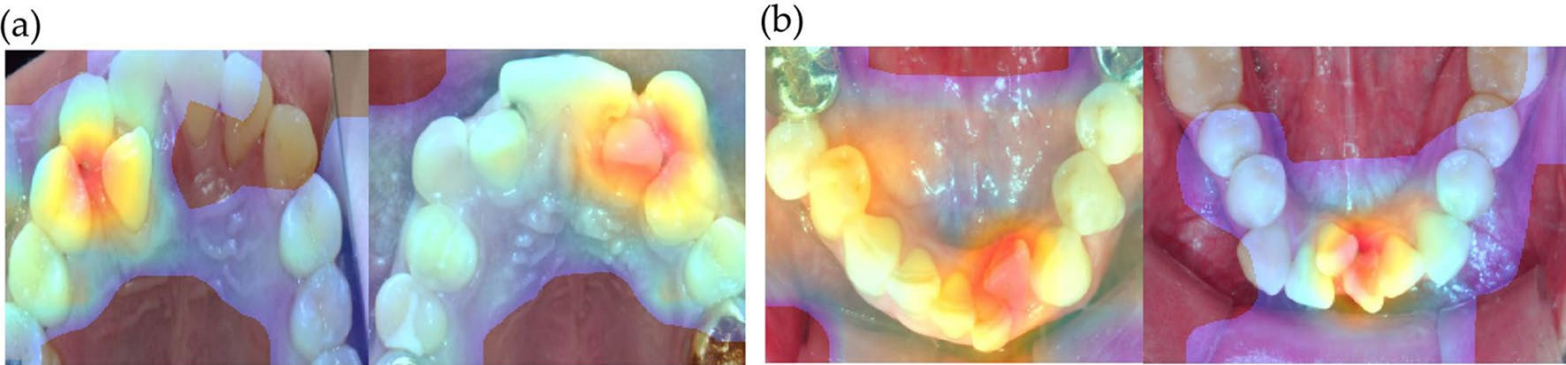
Grad-Cam examples. The red area indicates where reinforcing the AI models tends toward extraction. The signal is higher in the actual crowding area. (**A**) maxilla. (**B**) mandible.

In this way, this study suggests that artificial intelligence is capable of learning and digesting both qualitative and quantitative data, presenting the possibility of near-clinician-level diagnosis. This artificial intelligence can reproduce results similar to those of specialists and can help with clinical diagnosis. However, owing to the characteristics of deep learning, it is difficult to explain precisely how the machine can derive results^42^. Nevertheless, there might be patterns and consistency in recognizing and processing the image data as humans do because the results from AI models and clinicians were not significantly different. The Grad-CAM can be used to graphically highlight the region in the images that the decision-making AI models consider more important. As shown in Fig. 7, the signal in a similar area that clinicians consider important for deciding tooth extraction is amplified as a reddish tint. In other words, although the feeding data had no information regarding the crowding regions, the AI models themselves consider a high priority to these crowding regions to decide if extraction is more favorable.

The two models presented in the study, tooth landmark detection for crowding categorization and tooth extraction diagnosis through clinical intraoral photos, showed clinically sufficient accuracy to assist in real situations. However, caution should be taken when interpreting the results that, unlike other diseases with gold standards or definite criteria, such as infections, and hereditary diseases, the classification of dental crowding and tooth extraction as a treatment plan can be diverse among clinicians. This means that different doctors can diagnose one patient differently, and esthetic perspectives and cultural and ethnic conceptions can be accounted for. Likewise, there are “borderline cases” in which extraction and non-extraction can both be acceptable for satisfactory results by adapting adequate clinical skills and techniques^43^. This is because there is no single correct treatment plan, but more than one is possible. In such circumstances, artificial intelligence could aid clinicians in the comprehensive diagnosis, and the models can be adjusted in their favor by additional training if sufficient data are provided. In addition, by learning the ideas of famous and experienced experts, this AI approach could greatly assist young or new orthodontics dentists.

The following technical aspects should be considered: during the photo-taking procedure, the three-dimensional objects are optically projected onto a two-dimensional flat space, losing information about their unit length. Without exact magnification and scale information, it is impossible to retrieve the actual length of objects in millimeters from digital images. To overcome this problem, we converted the pixel-unit measurements of each tooth on the images to metric units by using statistical data from mean tooth size research^38^. Additionally, a study found that measurements on digital images are clinically reliable compared to direct measurements on stone models, that the errors were not statistically significant^44^. Another error arises from mirror angulations. When taking intraoral occlusal photographs, intraoral mirrors are used for a better view, which causes shortening of the teeth, varying with the mirror and tooth angulations. However, the photos used in this study were taken under clinical standards and formats by experts, enabling the attenuation of such errors to make results consistent^45^. Nevertheless, care should be taken when gathering images for AI models and interpreting their results.

Generally, VggNet showed a relatively better performance than ResNet, and models of deeper layers showed better results than shallower ones in the same network model group. VggNet is widely used because of its simple architecture and deep networks. However, the number of parameters is large because of the three fully connected layers. ResNet utilizes residual networks rather than plain networks, making it possible to overcome the degradation problem and deepen the network without a severe accuracy loss. However, because ResNet utilizes average pooling and has a small fully connected layer, the characteristics of partial image patterns can be eliminated or underrated. Thus, unlike image classification problems, the performance of landmark detection, which requires identifying specific detailed locations, might be relatively poor compared with VggNet, which has more fully connected layer parameters and conserves more characteristics of the image features.

The difficulty in taking mandibular occlusal photos as opposed to in the maxilla could be attributed to the insufficient patient’s mouth opening and improper tongue positioning. This results in inconsistent photo quality that eventually lead to lower accuracy of mandibular AI outputs. For example, if a patient fails to open their mouth sufficiently, an insufficient reflection angle will cause the labial surface of the mandibular teeth, especially the anterior teeth, to be enlarged. This results in poor quality and unwanted diversity of the dataset, thus lowering the performance.

This study had some limitations. First, samples of missing teeth and mixed and primary dentitions were excluded, which confined the validity of the models to patients with complete permanent dentitions. In addition, to analysis primary and mixed dentitions, different clinical methods and approaches are required. Second, the dataset was obtained from a single institution. If other clinics use different formats or angulations to take pictures, the resulting accuracy may differ. However, the photographs used for orthodontic recording were not taken arbitrarily, but with certain common clinical standards, and as such, there might not be much difference. This study aimed to suggest the capability of automated diagnosis in general and compare models, making it possible to use them in real clinics in the future. Finally, there may be several factors other than crowding that can influence the decision to extract a tooth, such as tooth inclination and lateral facial profile. In future research, it is necessary to develop a diagnostic model that encompasses all of these factors.

In conclusion, our study successfully constructed deep learning models for detecting tooth landmarks, classifying crowding categorization, and diagnosing the necessity of tooth extraction. In general, the performance of VggNet was better than that of ResNet, and the results were more accurate in the maxilla than in the mandible in terms of consistency. Overall, AI models with proper architecture and training can substantially help clinicians in orthodontic treatment planning.

## Data Availability

Data supporting the findings of the current study are available from the corresponding author upon reasonable request.
